# Nanoparticle-Based Biomaterials in Cancer Research: From Mechanistic Insights to Therapeutic Innovation

**DOI:** 10.3390/ijms27135930

**Published:** 2026-07-01

**Authors:** Manoochehr Rasekh, Sassan Hafizi

**Affiliations:** 1 College of Engineering, Design and Physical Sciences, Brunel University of London, Uxbridge UB8 3PH, UK; 2 School of Medicine, Pharmacy & Biomedical Sciences, University of Portsmouth, St. Michael’s Building, White Swan Road, Portsmouth PO1 2DT, UK

**Keywords:** cancer nanomedicine, biomaterials, nanoparticle drug delivery, tumour microenvironment, precision nanomedicine, cancer immunotherapy, stimuli-responsive nanoparticles, patient-derived organoids, artificial intelligence, theranostics

## Abstract

Cancer remains one of the most complex diseases to study and treat, with tumour microenvironment heterogeneity and therapeutic resistance continuing to limit clinical progress. Biomaterials-based nanoparticles have emerged as versatile platforms that not only advance understanding of cancer biology but also enable innovative therapeutic strategies. As mechanistic tools, nanoparticles can be used to investigate extracellular matrix interactions, mechanotransduction pathways, drug resistance, and tumour–immune crosstalk, providing insights into how physical and biochemical cues influence disease progression. Therapeutically, engineered nanoparticle systems have been developed for the targeted delivery of chemotherapeutics, nucleic acids, and immunomodulatory agents, incorporating features such as stimuli-responsive release, multifunctionality, and theranostic capabilities. Recent advances in patient-derived tumour models, high-throughput screening platforms, and artificial intelligence-assisted design are further driving the development of precision nanomedicine. Despite ongoing challenges related to biodistribution, safety, manufacturing scalability, and regulatory approval, nanoparticle-based biomaterials offer significant opportunities to bridge fundamental cancer research and clinical translation. This review highlights recent mechanistic and therapeutic advances, discusses key translational barriers, and outlines future directions at the interface of biomaterials, nanotechnology, and oncology.

## 1. Introduction

Cancer is a heterogeneous and dynamic disease driven not only by genetic and epigenetic alterations in malignant cells but also by complex interactions within the tumour microenvironment (TME). The TME, comprising extracellular matrix (ECM), stromal and immune cells, vasculature, and soluble factors, shapes tumour progression, metastasis, and therapeutic response, and contributes to spatial and temporal heterogeneity that undermines treatment efficacy. The complexity is important because features such as ECM remodelling, hypoxic gradients, and immune suppression create physical and biochemical barriers to effective therapy and promote adaptive resistance [1,2,3].

Biomaterials serve as dual-use platforms that both model these micro-environmental features in vitro and serve as engineered therapeutics in vivo. Engineered hydrogels, scaffolds, and nanoparticle probes enable controlled presentation of matrix composition, stiffness, and soluble cues to dissect mechanotransduction pathways (for example, YAP/TAZ signalling) and cell–cell/cell–matrix crosstalk under defined, tunable conditions. In parallel, biomaterial-based formulations (from polymers and liposomes to inorganic nanomaterials) are used therapeutically to improve drug solubility, bio-distribution, tumour penetration, and controlled release, effectively translating mechanistic insight into design rules for therapy [4,5,6,7].

Within this context, nanoparticles play a central role as multifunctional agents that bridge fundamental biology and clinical translation. Recent advances have expanded nanoparticle platforms (polymeric nanoparticles, liposomes, inorganic particles, dendrimers, and hybrid systems) and introduced stimuli-responsive designs that respond to pH, redox state, enzymes, or external triggers for on-demand drug release; alongside, theranostic constructs combine imaging and therapy to monitor delivery and response in real time. Nanoparticles are also being deployed to probe barrier function, model drug-resistance mechanisms, and modulate immune components of the TME, demonstrating their ability to elucidate mechanisms and improve therapeutic indices [8,9,10,11].

**Figure 1:**
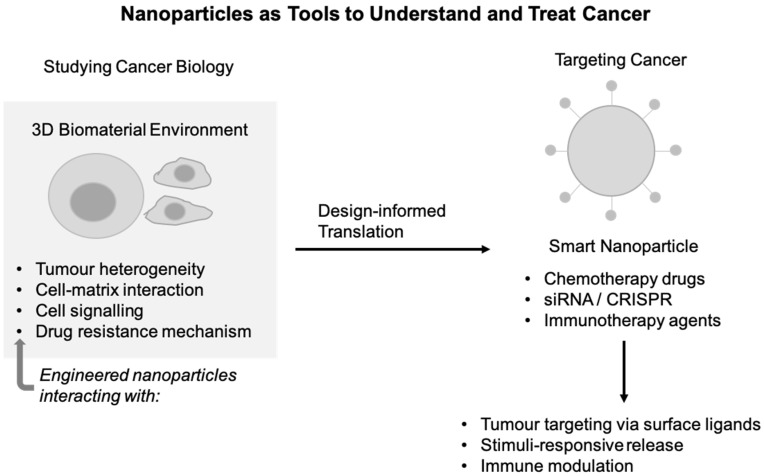
Schematic overview of nanoparticle-enabled cancer models and their translation to targeted therapeutic strategies.

This review examines nanoparticle-based biomaterials as both investigative and therapeutic tools in cancer research. The review first examines how biomaterial systems, particularly nanoparticles, have improved understanding of tumour biology, from ECM remodelling and mechanotransduction to drug resistance and immune regulation. It then discusses how these insights inform the design of advanced drug delivery systems, including targeted, stimuli-responsive, and multifunctional nanoparticles. Finally, it highlights emerging directions in precision nanomedicine, including patient-derived models and AI-assisted design, and addresses key translational and regulatory challenges for clinical translation of nano-therapeutics [12,13,14,15]. Nanoparticles serve as adaptable tools for modelling cancer biology and enabling targeted therapeutic delivery. In engineered three-dimensional biomaterial environments, nanoparticles and multicellular systems can be used to study tumour heterogeneity, cell–matrix interactions, cell signalling, and mechanisms of drug resistance. Findings from these biomimetic models inform the rational design of smart nanoparticles capable of delivering chemotherapeutics, gene-modulating cargos (e.g., siRNA or CRISPR components), and immunotherapeutic agents. These design strategies enable tumour targeting through surface ligands, stimuli-responsive cargo release, and modulation of immune responses (Figure 1).

## 2. Mechanistic Insights from Biomaterials

Beyond their therapeutic applications, biomaterials provide valuable platforms for investigating the biological and biophysical processes that drive tumour progression. In particular, nanoparticle-based systems enable the study of cancer cell interactions with the tumour microenvironment at high spatial and functional resolution. By serving as both experimental probes and model biomaterials, nanoparticles offer unique opportunities to elucidate the mechanisms underlying tumour development, heterogeneity, and therapy resistance.

### 2.1. Nanoparticles as Probes of Cancer Cells

Understanding cancer cell behaviour requires experimental systems capable of interrogating how cells sense, interpret, and respond to their surrounding microenvironment. In this section, we focus on how nanoparticles are increasingly used as tools for probing cancer cell–matrix interactions at length scales that are difficult to access using conventional biomaterial scaffolds alone. Because nanoparticles can be engineered with precise control over size, shape, stiffness, and surface chemistry, they provide an opportunity to dissect how physical and biochemical cues from the ECM regulate cancer cell adhesion, migration, and fate decisions [16,17].

One hallmark of tumour progression is the dynamic remodelling of the ECM, which alters matrix composition, ligand presentation, and mechanical properties [18]. NPs have been increasingly used to mimic or perturb these ECM features systematically, allowing researchers to isolate specific matrix cues and study their downstream effects on cancer cells. For example, NPs functionalised with ECM-derived ligands or adhesion peptides can engage integrin and other surface receptors, allowing systematic investigation of how ligand density, spatial organisation, and nanoscale presentation influence signalling pathways associated with proliferation, invasion, and epithelial–mesenchymal plasticity. In this context, NPs function not only as passive carriers, but as active probes that help clarify how cancer cells interpret their physical surroundings [19,20].

Beyond mimicking ECM interactions, nanoparticle uptake itself offers insight into cancer cell biology. Cellular internalisation pathways are highly sensitive to nanoparticle size, shape, charge, and surface properties, and these parameters can be tuned to interrogate endocytic mechanisms and intracellular trafficking routes. Studies using well-defined nanoparticle libraries have shown that subtle changes in surface chemistry can shift uptake among clathrin-mediated, caveolar, or macropinocytic pathways, with important consequences for intracellular signalling and drug response. These findings show how nanoparticle–cell interactions can be leveraged to better understand heterogeneity in cancer cell uptake behaviour, including differences between treatment-sensitive and -resistant populations [21].

Together, these approaches position NPs as mechanistic tools for studying cancer cell-ECM interactions and uptake processes at the nanoscale. By linking tunable material properties with quantitative biological readouts, nanoparticle-based probes help bridge simplified in vitro models and the complexity of the tumour microenvironment. The following sections build on these mechanistic insights to discuss how similar design principles are translated into nanoparticle-based therapeutic strategies for targeted drug delivery and cancer treatment [22,23]. To emphasise NPs as mechanistic probes rather than solely as delivery vehicles, Table 1 summarises representative nanoparticle systems and the biological processes they are used to investigate. By varying physicochemical properties such as size, surface chemistry, and ligand presentation, these platforms enable controlled investigation of cancer cell-ECM interactions and uptake pathways. Together, these studies link nanoparticle design to specific mechanistic insights into tumour cell behaviour.

### 2.2. Biophysical and Biochemical Cues

This section examines how biophysical and biochemical cues within the tumour microenvironment, particularly matrix stiffness and viscoelasticity, and how these cues shape cancer cell behaviour and disease progression. Solid tumours are mechanically distinct from normal tissues, with elastic moduli increasing from ~0.1–1 kPa in healthy epithelial tissue to ~5–50 kPa or higher in tumours, largely due to collagen accumulation and enzymatic crosslinking [27,28]. These mechanical changes are now recognised as active regulators of tumour growth rather than passive by-products of malignancy [27].

Cancer cells sense matrix stiffness through integrin-based adhesions and associated mechanotransduction pathways, which couple extracellular forces to cytoskeletal tension and downstream signalling. Increased stiffness promotes focal adhesion maturation, activation of focal adhesion kinase (FAK) and RhoA/ROCK signalling, and enhanced actomyosin contractility, leading to nuclear localisation of transcriptional regulators such as YAP/TAZ [27,29]. This marks a shift toward a more aggressive, therapy-resistant cancer phenotype. Several studies summarised in recent reviews report that YAP/TAZ nuclear translocation is commonly observed when substrate stiffness exceeds ~5–10 kPa, a range found in fibrotic and tumour-associated matrices [27,29]. These mechano-sensitive pathways reinforce malignant phenotypes, including increased survival, proliferation, migration, invasiveness and drug resistance.

In addition to elastic stiffness, matrix viscoelasticity, the ability of tissues to relax stress over time, has emerged as an important but less explored regulator of cancer cell behaviour. Viscoelastic matrices with stress-relaxation times on the order of seconds to minutes enable cells to remodel their surroundings more efficiently, leading to increased spreading, traction, and mechano-sensitive gene expression compared with purely elastic substrates [29,30]. Recent studies suggest that viscoelasticity modulates mechanotransduction independently of stiffness, affecting nuclear deformation and transcriptional programmes linked to migration and plasticity [30].

**Figure 2:**
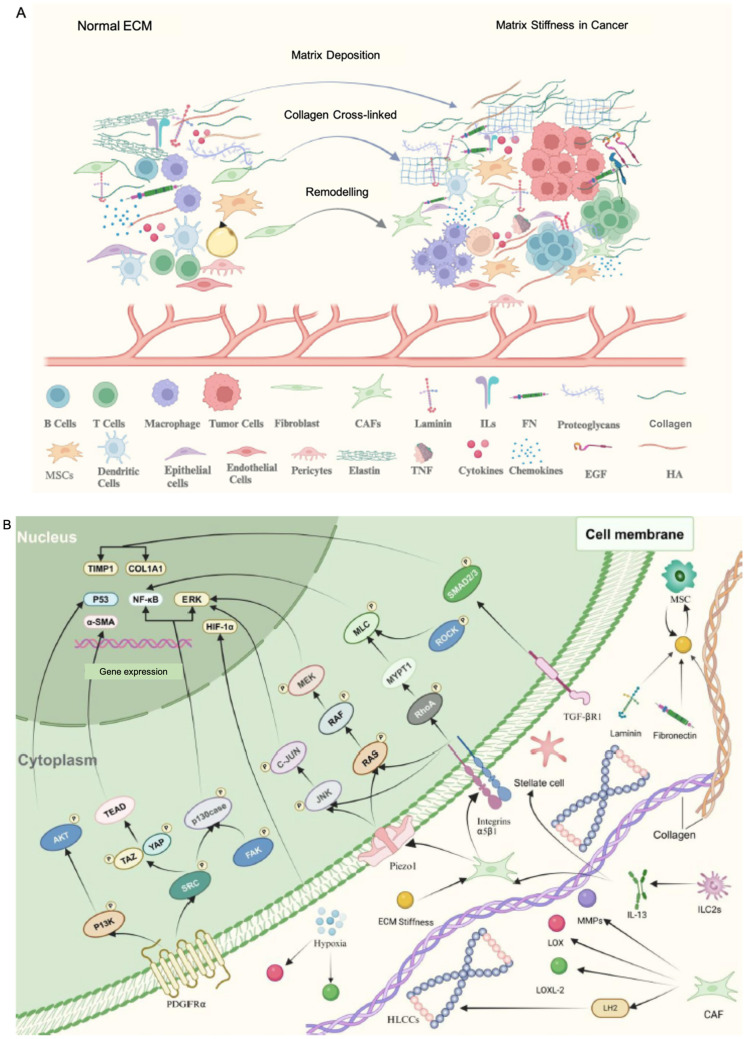
Extracellular matrix stiffening and mechanotransduction in the tumour microenvironment. (**A**) Comparison of ECM composition in normal tissue and tumours, highlighting increased matrix deposition in the form of collagen and hyaluronic acid accumulation, cytokine- and TGFβ-driven remodelling, and LOX-mediated collagen crosslinking that contribute to matrix stiffening and reinforce tumour–stroma interactions. (**B**) ECM stiffness-activated mechanotransduction pathways in tumour cells, in which mechanical cues are sensed by receptors such as integrins, Piezo channels, and PDGFR, leading to activation of intracellular signalling pathways mediated by FAK/SRC, ERK, AKT, β-catenin, RhoA–ROCK, and YAP/TAZ, with stromal cells and hypoxia further amplifying stiffness-associated phenotypes [33].

ECM remodelling further links mechanical and biochemical signalling in tumours. Cancer and stromal cells secrete matrix metalloproteinases (MMPs) and crosslinking enzymes such as lysyl oxidase (LOX), contributing to increasing local matrix stiffness several-fold and generating spatially heterogeneous mechanical niches within tumours [27,28,31]. These remodelling-associated regions often coincide with invasive fronts and are associated with impaired drug penetration, activation of mechano-sensitive survival pathways, and altered immune cell infiltration [28,31]. These observations highlight the importance of incorporating both mechanical and biochemical ECM cues into biomaterial and nanoparticle-based models aimed at probing tumour biology and therapeutic response [32]. Increased collagen and hyaluronic acid, TGFβ-mediated remodelling, and LOX-driven collagen crosslinking collectively increase matrix stiffness. Tumour and stromal cells sense these mechanical changes through integrins, Piezo channels, and PDGF receptor (PDGFR), activating downstream signalling pathways including FAK/SRC, RhoA-ROCK, and YAP/TAZ. Figure 2 summarises key ECM components and mechanotransduction pathways [33].

### 2.3. Tumour Heterogeneity and Therapy Resistance

Tumour heterogeneity arises from spatial and temporal variations in cell phenotype, genetic profile, and microenvironmental conditions, creating gradients in oxygen, nutrients, pH, and drug exposure that strongly influence therapeutic outcomes. One of the key microenvironmental gradients in solid cancers is hypoxia, driven by abnormal vasculature that cannot meet the metabolic demands of rapidly proliferating tumour cells. Hypoxic regions are known to promote angiogenesis, metastasis, and resistance to chemotherapy, radiotherapy, and immunotherapy through the activation of transcriptional programmes such as hypoxia-inducible factors (HIFs), which regulate metabolism, apoptosis, and stemness pathways. Recent studies highlight that hypoxia contributes not only to drug resistance but also to immune suppression and metabolic shifts that are tightly interwoven with intratumoural heterogeneity and poor prognosis in patients [34,35].

Biochemical gradients within tumours, including oxygen and nutrient depletion, interact with physical barriers such as a dense ECM and elevated interstitial pressure to limit drug penetration and create heterogeneous therapeutic access across tumour regions. These barriers contribute to subpopulations of cells that are shielded from conventional therapies and can act as reservoirs for relapse. For instance, in pancreatic ductal adenocarcinoma, dense desmoplastic stroma and poor perfusion combine to create profound hypoxia and drug exclusion that underlie resistance to chemotherapy and radiotherapy [36]. Emerging work emphasises hypoxia-responsive nanocarrier systems, such as bio-reductively cleavable linkers (e.g., nitro-imidazole or azobenzene-based linkers) and hypoxia-activated prodrugs, which can selectively release payloads in low-oxygen regions, directly addressing these gradients. Other strategies aim to overcome these barriers through surface functionalisation with tumour-targeting ligands (e.g., aptamers and antibodies), hypoxia-activated prodrugs that become cytotoxic under low oxygen conditions, and multi-stimuli-responsive designs integrating pH, enzymatic, or exogenous triggers [35,37].

Tumour heterogeneity also influences nanoparticle uptake and intracellular fate of nanoparticles themselves, an important consideration when using NPs as both probes and therapeutic carriers. Experimental studies show that hypoxia can alter nanoparticle endocytosis and exocytosis dynamics in cancer cells, suggesting that oxygen gradients and phenotypic diversity modulate nanoparticle access and retention. In breast cancer models, hypoxia increases nanoparticle internalisation and recycling, reflecting a complex interplay between micro-environmental conditions and cellular trafficking machinery. These findings show that heterogeneity in oxygenation and metabolic state can shape nanoparticle behaviour and complicate uniform delivery, even within a single tumour [38].

Nanoparticle-based tools are increasingly being developed to study and overcome resistance mechanisms driven by tumour heterogeneity. Beyond hypoxia-triggered drug release, nanoparticles can also deliver oxygen or oxygen-generating agents, normalise tumour vasculature, or target HIF-mediated signalling pathways to counteract hypoxia-driven resistance. Such strategies aim to convert hostile microenvironmental gradients into therapeutic vulnerabilities by enhancing drug distribution, modulating local metabolism, and disrupting adaptation pathways that sustain resistant niches. The integration of these mechanistic insights with smart nanoparticle design may help reduce heterogeneity-associated resistance and improve therapeutic predictability [39,40].

Nanoparticles are increasingly used in cancer therapy as drug carriers that can improve safety and efficacy over conventional approaches. Compared with traditional drug carriers, NPs are often well tolerated, circulate longer, and can be engineered to remain stable while releasing their payload in a controlled manner. These properties allow nanoparticles to improve drug delivery and, in some cases, enhance immune responses against tumours. As a result, nanoparticle-based systems are increasingly explored in cancer immunotherapy, particularly because they can persist in tumours for extended periods and be tailored to target specific cells or tissues. A major challenge in cancer treatment is the TME, which strongly influences tumour growth and therapy response. In addition to cancer cells, the TME contains many non-cancerous cells, including immune cells (such as macrophages, natural killer cells, and T cells), fibroblasts, dendritic cells, and adipocytes. Together, these components create an immunosuppressive environment that limits the effectiveness of immunotherapies. Poor blood flow, abnormal lymphatic drainage, and hypoxia further reduce drug penetration and immune activity [40,41].

Nanoparticles offer opportunities to address these barriers by targeting key components of the tumour microenvironment. Tumour growth and abnormal vasculature promote the accumulation of immunosuppressive cells, such as myeloid-derived suppressor cells (MDSCs) and regulatory T cells, which secrete factors like vascular endothelial growth factor (VEGF) and TGF-β and contribute to hypoxia and immune evasion [42]. Several nanoparticle systems have been shown to selectively target these cells or signalling molecules, helping to reprogram the TME toward a more immune-active state and restore sensitivity to therapy. NPs preferentially accumulate in tumour tissue due to leaky blood vessels and impaired lymphatic drainage, a phenomenon often referred to as the enhanced permeability and retention (EPR) effect. Their surfaces can be modified with targeting ligands (e.g., antibodies or peptides) to further improve specificity and therapeutic efficiency. Together, these features make NPs versatile tools for overcoming microenvironment-related resistance and enhancing the overall effectiveness of cancer immunotherapy [23,40]. Nanoparticle-based drug delivery systems rely on multiple targeting mechanisms to achieve selective accumulation in tumour tissues. These mechanisms include passive targeting through the EPR effect, active targeting via ligand–receptor interactions, and stimuli-responsive strategies triggered by tumour-specific biochemical conditions. However, it is important to note that the EPR effect is highly heterogeneous and often inefficient in clinical settings. Its magnitude varies significantly across tumour types, patients, anatomical sites, vascular architecture, stromal density, and prior treatments. While the EPR effect has been well demonstrated in preclinical tumour models, its translational predictability in human cancers remains limited. Therefore, passive accumulation alone is generally insufficient for consistent tumour targeting and is often complemented by active targeting and microenvironment-responsive strategies to improve delivery efficiency. This limitation has been increasingly recognised as a major translational barrier in nanomedicine.

Also, NPs provide a platform to investigate immune cell modulation within tumours. Engineered NPs enable controlled delivery of immunomodulatory agents, reporters, or inhibitors to specific immune cell populations, allowing researchers to study how signalling pathways in macrophages, dendritic cells, and T cells are altered in tumours. Unlike systemic administration, nanoparticle-based approaches can localise immune effects spatially and temporally, helping clarify how immune suppression is established and maintained in heterogeneous tumour niches [41]. NPs have been particularly valuable for studying immunosuppressive pathways mediated by tumour-associated macrophages (TAMs) and dysfunctional T cells, which are major barriers to effective anti-tumour immunity. For example, nanoparticle systems have been designed to preferentially accumulate in TAMs and modulate polarisation states, revealing links between macrophage phenotype, cytokine production, and T cell exhaustion [43]. Similarly, nanoparticle-mediated delivery of immune checkpoint regulators or metabolic modulators has enabled precise interrogation of suppressive signalling circuits in T cells, providing insights into how immune dysfunction arises and how it may be reversed. Together, these approaches highlight the utility of NPs not only as therapeutic carriers but also as experimental tools for unravelling tumour–immune interactions.

**Figure 3:**
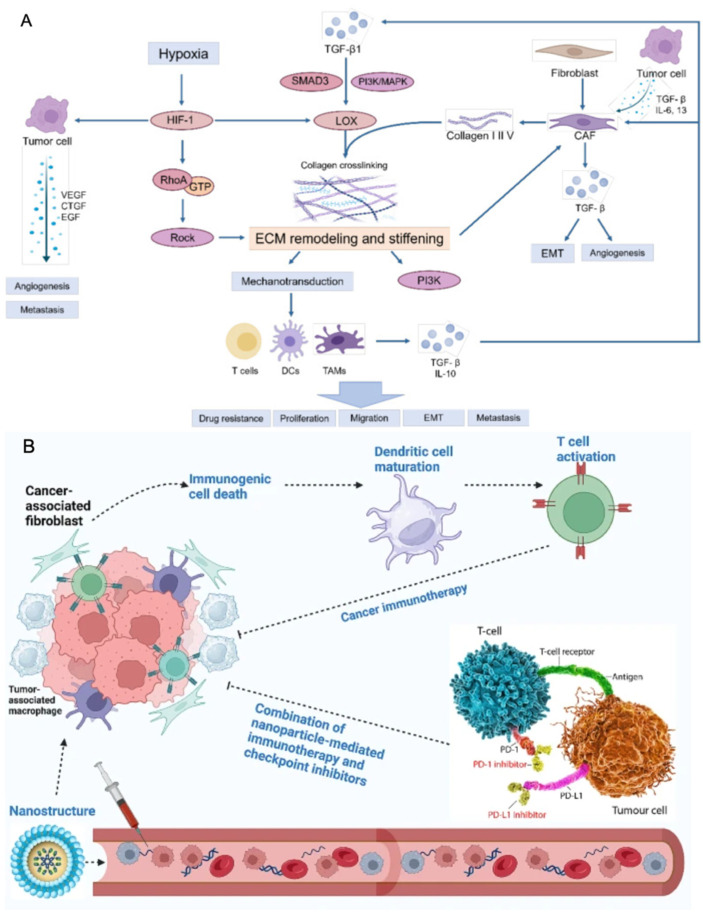

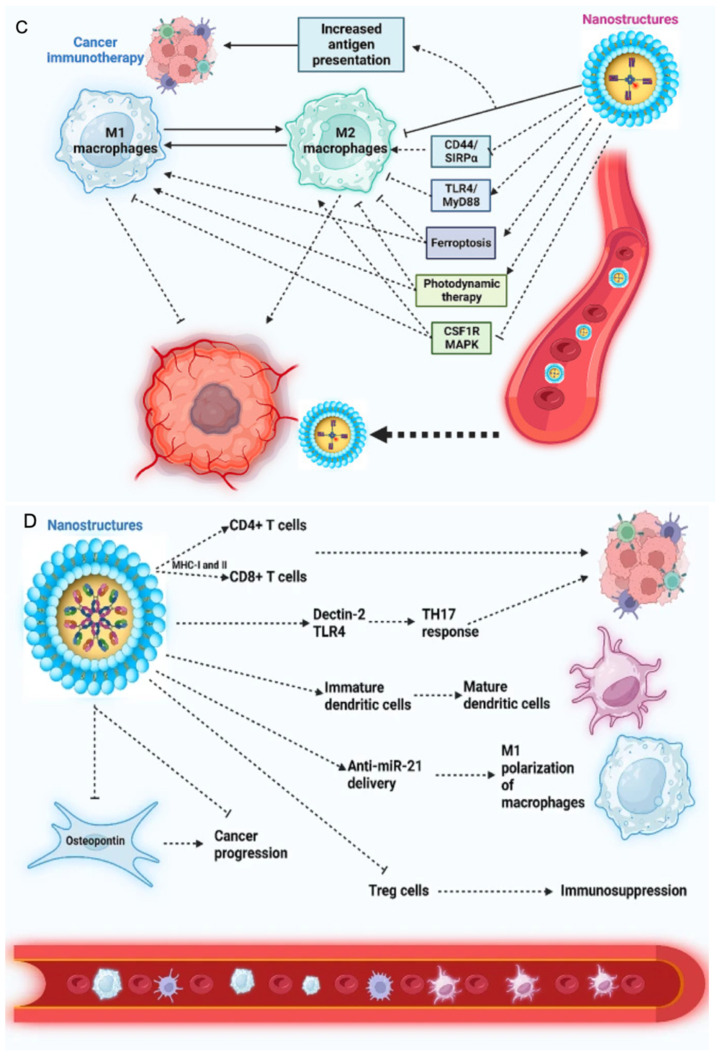
Physical and immune barriers within the tumour microenvironment and their interaction with nanoparticle-based interventions. (**A**) Schematic overview of the primary contributors to extracellular matrix stiffening in the tumour microenvironment and the resulting mechanical changes, such as solid stress and altered tissue organisation [44]. (**B**) Cellular composition of the tumour microenvironment, highlighting immunosuppressive immune populations and stromal interactions. (**C**) Illustrative example of nanoparticle-mediated reprogramming of tumour-associated macrophages to enhance antitumour immune activity. (**D**) Nanoparticle-based modulation of immune signalling pathways to promote T cell activation and overcome immune resistance [41].

Tumour heterogeneity and therapeutic resistance arise from the combined influence of physical constraints and immune dysfunction within the tumour microenvironment. Dense extracellular matrices and abnormal mechanical forces restrict the transport and distribution of therapeutic agents, including NPs, while immunosuppressive immune populations further limit effective anti-tumour responses. NP-based systems provide versatile platforms to probe these barriers and, in some contexts, to modulate them by navigating heterogeneous tumour regions, improving penetration within stiff matrices, and preferentially accumulating in immunosuppressive niches. In particular, some nanoparticle strategies have been shown to reprogram TAMs from an immunosuppressive (M2-like) phenotype to a pro-inflammatory (M1-like) state, thereby enhancing immune activity and therapeutic responsiveness. Figure 3 summarises these mechanical, immune, and delivery-related challenges and how NP-based approaches interface with the tumour microenvironment [31,41,44]. Together, these mechanistic insights demonstrate how nanoparticle-based systems can both interrogate and influence key processes within the tumour microenvironment. Understanding these interactions provides a foundation for the rational design of therapeutic nanoparticles aimed at improving drug delivery, overcoming resistance mechanisms, and enhancing treatment efficacy, as discussed in the following section.

Table 2 provides a concise summary of representative nanoparticle strategies used to investigate and address major sources of tumour heterogeneity and therapy resistance discussed in this section.

## 3. Nanoparticle-Based Cancer Therapeutics

Building on the mechanistic insights discussed above, nanoparticle-based drug delivery systems have emerged as an important strategy for improving cancer treatment. Conventional chemotherapy is often limited by poor specificity, rapid drug degradation, and systemic toxicity that damages healthy tissues. Nanoparticles can help address these limitations by encapsulating therapeutic agents and improving their stability, circulation time, and accumulation in tumour tissues. Their small size and tunable surface properties allow controlled interactions with biological systems in controlled ways, making them attractive carriers for anticancer drugs, nucleic acids, and imaging agents. As a result, nanoparticle platforms are increasingly used to enhance drug delivery efficiency while reducing off-target effects in cancer therapy [50].

A key advantage of nanoparticle systems is that they can be engineered with different materials and functionalities depending on the therapeutic goal. These platforms can be designed to improve drug solubility, control drug release, and enable selective delivery to tumour tissues. In addition, nanoparticle systems can integrate multiple functions, such as drug delivery, imaging, and immune modulation, within a single platform. This flexibility has enabled the development of diverse nanocarriers that address several challenges associated with cancer therapy, including drug resistance, poor tissue penetration, and heterogeneous tumour environments [50].

### 3.1. Nanoparticle Platforms and Targeting Strategies

Several nanoparticle types have been developed as drug delivery carriers in cancer therapy. Liposomes are classical phospholipid bilayer vesicles that resemble biological membranes and can encapsulate both hydrophilic and hydrophobic drugs, which has led to their widespread use in clinical formulations. However, it is important to distinguish these conventional liposomes from modern ionisable lipid nanoparticles (LNPS), which are specifically engineered for nucleic acid delivery. Polymeric nanoparticles, typically made from biodegradable polymers such as poly (lactic-co-glycolic acid) (PLGA) or poly (lactic acid) (PLA), allow controlled drug release and can be engineered to respond to environmental stimuli. Dendrimers are highly branched macromolecules with numerous surface functional groups that enable precise drug loading and molecular modification. Inorganic nanoparticles, such as gold or silica nanoparticles, offer unique physical properties that can support imaging, photothermal therapy, or controlled drug delivery [51,52,53]. Among different nanocarrier systems, liposomes and polymeric nanoparticles are the most widely studied for cancer therapy. Liposomes consist of phospholipid bilayers that protect encapsulated drugs from degradation and improve their pharmacokinetic profiles. In contrast, ionisable lipid nanoparticles rely on pH-triggered charge modulation rather than stable bilayer encapsulation, enabling superior intracellular delivery of genetic material. Polymeric nanoparticles, on the other hand, provide greater structural flexibility and can be designed to release drugs gradually or in response to specific stimuli such as pH or temperature. These features make both systems valuable for improving the therapeutic index and reducing systemic toxicity compared with conventional chemotherapy [54]. In addition to classical liposomes, ionisable LNPs have emerged as a clinically validated and highly efficient lipid-based platform for nucleic acid delivery. Unlike conventional liposomes, LNPs are composed of ionisable lipids, helper phospholipids, cholesterol, and PEG-lipids, which together enable efficient encapsulation of RNA cargo, colloidal stability, and in vivo delivery. A key distinguishing feature of LNPs is their pH-responsive ionisation behaviour, which facilitates endosomal escape after cellular uptake, significantly enhancing cytosolic delivery of nucleic acids such as siRNA and mRNA. This mechanism is fundamentally different from classical liposomal drug encapsulation and positions LNPs as a leading platform in gene-based cancer therapeutics and clinically approved RNA delivery systems.

#### Emerging Gene Delivery Platforms

Beyond conventional nanoparticle systems, several advanced delivery platforms have emerged for nucleic acid and gene-based cancer therapies. Among these, LNPs, extracellular vesicles (EVs), and viral vectors represent the most clinically advanced approaches. Ionisable LNPs have become the leading non-viral platform for the delivery of mRNA, siRNA, and gene-editing cargo owing to their efficient encapsulation capacity, scalable manufacturing, and ability to promote endosomal escape. Extracellular vesicles, including exosomes, offer a biologically derived alternative with inherent biocompatibility, low immunogenicity, and natural cell-targeting properties, making them attractive candidates for RNA and protein delivery. Viral vectors, particularly adeno-associated viruses (AAVs), remain highly efficient gene transfer vehicles and have demonstrated long-term transgene expression in several clinical applications. However, each platform presents distinct translational challenges, including immune recognition, manufacturing complexity, cargo limitations, biodistribution control, and safety considerations.

Recent comparative analyses suggest that no single delivery system is universally optimal; rather, platform selection depends on therapeutic objectives, payload characteristics, target tissues, and clinical requirements. Increasingly, hybrid approaches that combine the scalability of synthetic nanoparticles with the biological targeting capabilities of natural or viral systems are being explored to enhance delivery precision and therapeutic efficacy. These developments highlight the evolving landscape of gene delivery technologies and their growing relevance to cancer nanomedicine [55].

Dendrimers and inorganic nanoparticles offer additional advantages for targeted cancer therapy. The highly branched structure of dendrimers provides a large number of surface functional groups that can be used to attach drugs, imaging agents, or targeting ligands. This structural precision enables controlled drug loading and improved interactions with tumour cells. Inorganic nanoparticles, including gold and mesoporous silica nanoparticles, are valued for their large surface area and unique optical or magnetic properties, which support combined therapeutic and diagnostic applications, often referred to as theranostics [53,56].

To further enhance tumour specificity, nanoparticles are often modified with targeting molecules on their surface. These ligands can include antibodies, peptides, small molecules, or aptamers that bind to receptors overexpressed on cancer cells. Such surface functionalisation enables active targeting, allowing nanoparticles to recognise and bind tumour cells more efficiently than non-targeted carriers. For example, aptamers and monoclonal antibodies have been widely used to improve the selectivity of nanoparticle uptake in cancer cells by recognising tumour-associated biomarkers. These strategies may increase drug accumulation in tumour tissues while minimising damage to healthy organs [57,58]. Nanoparticle platforms used for cancer therapy differ in their structural properties, drug-loading capacity, and targeting potential. Major nanocarrier classes, including liposomal, polymeric, dendrimer-based, and inorganic nanoparticles, offer distinct advantages for drug delivery and tumour targeting. Table 3 summarises key nanoparticle platforms, their main characteristics, and representative applications in cancer therapy.

In addition to the material composition of nanoparticles, surface functionalisation plays an important role in determining their biological performance. By attaching targeting ligands to the nanoparticle surface, nanocarriers can recognise specific receptors that are overexpressed on tumour cells or within the tumour microenvironment. These targeting strategies can improve tumour accumulation and enhance cellular uptake. Table 4 summarises commonly used targeting ligands and their corresponding tumour-associated receptors.

Recent studies show that surface-functionalised nanoparticles can significantly improve the selective delivery of anticancer agents to tumour cells. One widely used strategy involves folic acid as a targeting ligand because the folate receptor is frequently overexpressed in several cancers, including ovarian, breast, and glioblastoma. For example, folate-modified gold nanoparticles loaded with the anti-inflammatory drug indomethacin showed enhanced uptake in folate receptor-positive glioblastoma cells and spheroids. In this study, the targeted nanoparticles increased cytotoxicity by more than thirty-fold compared with the free drug and significantly inhibited tumour cell proliferation while having minimal effects on receptor-negative cells. These findings show how ligand-directed targeting can improve therapeutic selectivity and efficacy in cancer treatment [65,66].

Other studies have explored combining multiple targeting ligands to enhance nanoparticle specificity. In a recent study, silica nanoparticles functionalised with both hyaluronic acid and folic acid were designed to target CD44 and folate receptors simultaneously in colon cancer cells. The study demonstrated that cellular targeting efficiency depended strongly on ligand density and receptor expression levels. Nanoparticles with optimised ligand ratios showed significantly greater binding and uptake in receptor-positive tumour cells compared with control cell lines, showing how nanoparticle surface chemistry can be tuned to improve targeting performance. These results suggest that rational design of ligand density and receptor specificity is critical for achieving efficient nanoparticle-mediated drug delivery [71,72,73].

Targeting ligands have also been incorporated into multifunctional nanoparticle systems to combine therapeutic modalities. For instance, dual-targeted magnetic nanoparticles carrying both folic acid and transferrin were developed for retinoblastoma treatment. These nanoparticles delivered vincristine while simultaneously enabling magnetic hyperthermia therapy under an alternating magnetic field. Experimental results showed greater cytotoxicity in tumour cells compared with non-targeted systems, as well as increased reactive oxygen species generation and reduced colony formation in cancer cells. The combination of receptor-mediated targeting and magnetic activation produced stronger anticancer effects than chemotherapy or hyperthermia alone, demonstrating the potential of multifunctional nanocarriers for improving therapeutic outcomes [74].

**Figure 4:**
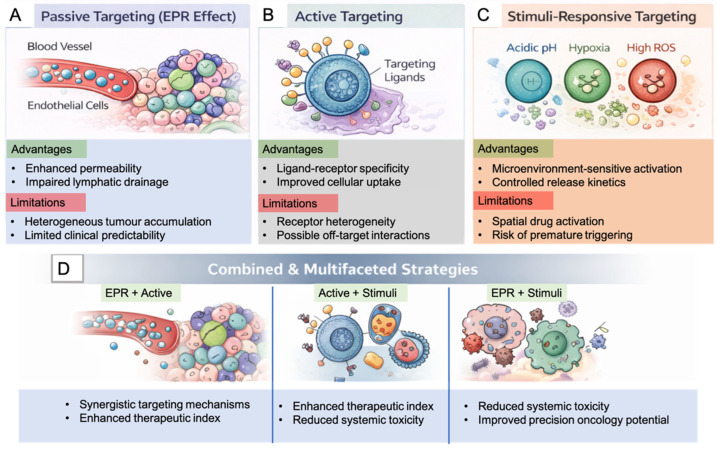
Targeting strategies for nanoparticle-based cancer therapy. Schematic illustration of major nanoparticle targeting mechanisms, including (**A**) passive targeting through tumour vascular permeability, (**B**) active targeting via ligand–receptor interactions, (**C**) stimuli-responsive activation triggered by tumour microenvironment cues, and (**D**) cellular or immune-mediated targeting pathways that enhance tumour localisation and therapeutic delivery. Adapted from [75]. ROS, reactive oxygen species (e.g., superoxide, hydrogen peroxide) associated with oxidative stress in the tumour microenvironment; EPR, enhanced permeability and retention.

Nanoparticle-based drug delivery systems rely on multiple targeting mechanisms to achieve selective accumulation in tumour tissues. These mechanisms include passive targeting through the enhanced permeability and retention effect, active targeting via ligand–receptor interactions, and stimuli-responsive strategies triggered by tumour-specific biochemical conditions. Together, these approaches are intended to improve nanoparticle localisation, cellular uptake, and therapeutic efficacy. Figure 4 illustrates major targeting strategies used in nanoparticle-based cancer therapy [75].

### 3.2. Stimuli-Responsive and Smart Delivery Systems

A major challenge in cancer drug delivery is achieving selective drug release at the tumour site while minimising systemic toxicity. Stimuli-responsive nanoparticles address this challenge by exploiting intrinsic features of the tumour microenvironment, such as acidic pH and elevated redox potential, to achieve selective drug release. For example, dual pH/redox-responsive nanogels loaded with doxorubicin have demonstrated enhanced drug release under acidic and glutathione-rich conditions, leading to significantly higher cytotoxicity in colon cancer cells compared with free drug [76]. In parallel, redox-responsive nanoparticles incorporating disulfide linkages have been widely developed to exploit intracellular glutathione levels, enabling triggered drug release within tumour cells and improving therapeutic efficacy while reducing systemic toxicity [77].

Beyond pH and redox cues, enzyme-responsive systems can further enhance specificity by responding to tumour-associated proteases, while externally triggered platforms provide greater spatial and temporal control. For instance, hybrid nanocarriers designed to respond to pH, redox conditions, and enzymatic activity have shown improved intracellular delivery and controlled drug release across biological barriers [78]. In addition, thermally or externally activated systems such as hyperthermia-assisted delivery have demonstrated improved tumour accumulation and drug release, demonstrating the benefit of combining endogenous and exogenous triggers to enhance treatment outcomes [78]. More advanced systems integrate multiple stimuli to address tumour heterogeneity and enable on-demand drug release. Dual-responsive nanoparticles combining pH and redox sensitivity have shown enhanced tumour targeting, increased cellular uptake, and synergistic therapeutic effects when combined with phototherapy modalities [79]. Similarly, redox and ROS-responsive platforms exploit tumour-specific oxidative stress to selectively trigger drug release, improving intracellular drug concentration and therapeutic outcomes [80]. These approaches demonstrate the potential of multi-stimuli systems for precise and adaptable cancer therapy.

### 3.3. Multifunctional and Combination Nanoparticles

Multifunctional nanoparticles that integrate diagnostic and therapeutic functions, commonly referred to as theranostic systems, have gained significant attention in cancer research. Redox-responsive nano-platforms, for example, have been developed to combine imaging capabilities with controlled drug delivery, enabling real-time monitoring of treatment while simultaneously enhancing therapeutic efficacy [81]. In addition, inorganic and hybrid nanomaterials can support multimodal imaging and therapy, providing insight into nanoparticle distribution and treatment response, which is important for precision medicine approaches. For example, gold and iron oxide nanoparticles have been widely explored for combined imaging (e.g., MRI, CT, or photoacoustic imaging) and therapy, including photothermal and drug-based approaches. In one study, gold nano-rods functionalised with chemotherapeutic agents enabled simultaneous tumour imaging and photothermal ablation, resulting in enhanced tumour reduction compared with monotherapy. Such platforms provide insight into nanoparticle distribution and therapeutic response, supporting the development of more personalised treatment strategies [82,83,84,85].

Co-delivery strategies using nanoparticles enable the simultaneous delivery of multiple therapeutic agents to address tumour complexity. For instance, dual-responsive nanocarriers capable of delivering chemotherapeutic drugs alongside other therapeutic modalities have demonstrated improved tumour suppression through synergistic therapeutic effects [79]. Similarly, multi-functional nanocarriers designed for combined chemotherapy and stimuli-responsive release have shown enhanced intracellular delivery and improved anticancer efficacy compared with single-agent systems [76]. Maruf et al. (2022) [76] developed pH and redox-responsive nanogels for doxorubicin delivery that remain stable under physiological conditions but release the drug efficiently in tumour-like environments. These nanocarriers exhibited high drug loading capacity and enhanced release under acidic and glutathione-rich conditions, resulting in significantly improved cytotoxicity in HCT116 colon cancer cells compared with free doxorubicin. This study demonstrates the potential of dual-responsive nanogels to improve drug stability during circulation while enabling selective tumour-targeted therapy [76]. These findings show the importance of coordinated delivery in maximising therapeutic outcomes.

Furthermore, nanoparticle-based systems offer strategies to overcome multidrug resistance (MDR), a major limitation in cancer therapy. Redox-responsive nanocarriers can increase intracellular drug accumulation by releasing drugs directly within tumour cells, thereby bypassing resistance-associated efflux mechanisms [77]. In addition, redox-manipulating nanomedicines can modulate intracellular oxidative balance, enhancing drug sensitivity and improving therapeutic outcomes in resistant tumours [86]. These approaches show how multifunctional nanoparticle systems can address multiple resistance pathways simultaneously. Table 5 provides a comparative overview of these approaches, highlighting their design principles, activation mechanisms, and representative therapeutic outcomes. In addition to improving drug delivery and overcoming resistance mechanisms, nanoparticle platforms can be engineered to modulate immune responses within the tumour microenvironment, further broadening their role in cancer therapy.

## 4. Immunomodulatory Biomaterial-Based Nanoparticles for Cancer Therapy

An important extension of these multifunctional capabilities is the use of biomaterial-based nanoparticles to deliver immunomodulatory agents directly to specific immune cell populations within the tumour microenvironment. By tuning surface chemistry and incorporating targeting ligands, these systems can preferentially accumulate in TAMs, dendritic cells, and other immune subsets, enabling localised delivery of cytokines, antigens, or immune checkpoint inhibitors. This targeted delivery can improve therapeutic efficacy while minimising systemic toxicity and off-target immune activation. For example, nanoparticle systems have been shown to enhance uptake by dendritic cells and promote antigen presentation, thereby enhancing T cell-mediated immune responses [41,88].

Beyond targeted delivery, nanoparticles can remodel the tumour immune microenvironment by reversing immunosuppressive conditions. Many tumours are dominated by suppressive immune populations such as M2-like TAMs, regulatory T cells (Tregs), and MDSCs, which suppress effective anti-tumour immunity. Nanoparticle-based strategies have been shown to promote macrophage repolarisation toward a pro-inflammatory (M1-like) phenotype, enhance dendritic cell maturation, and increase cytotoxic T cell infiltration. These changes can convert immunologically ‘cold’ tumours into ‘hot’ immune-responsive states, improving the effectiveness of immunotherapy [41,89].

**Figure 5:**
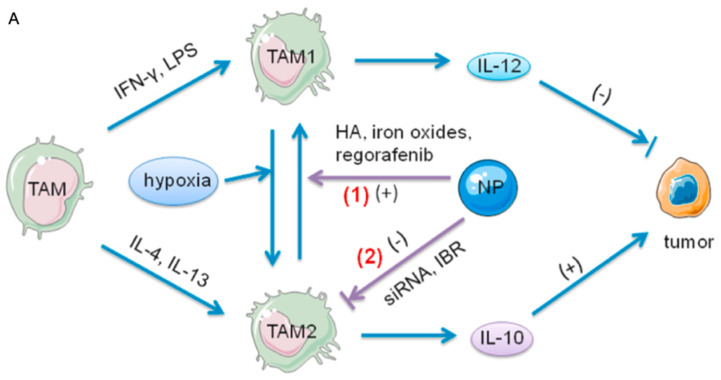

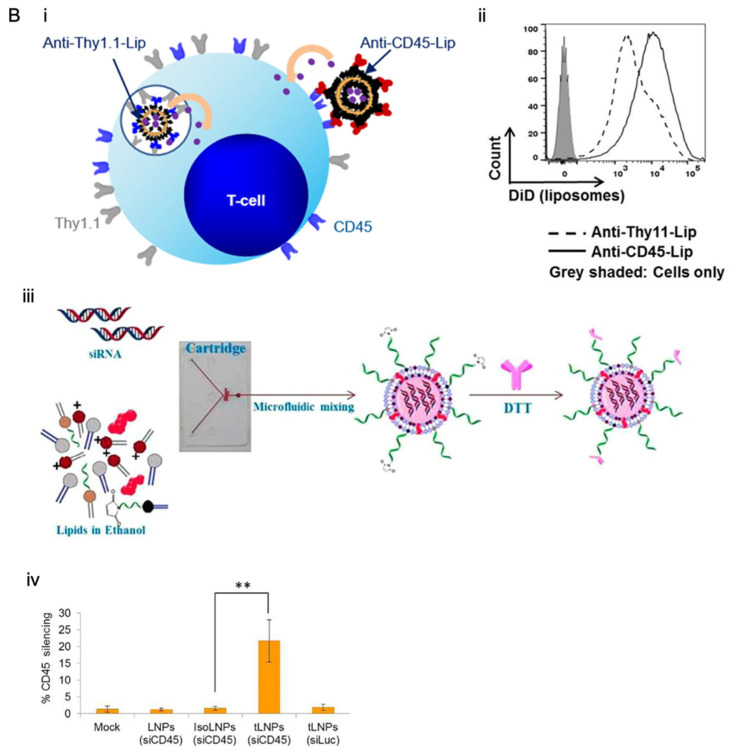
Nanoparticle-mediated immune modulation in the tumour microenvironment. (**A**) Macrophage polarisation states (M1 and M2) and nanoparticle-driven M2-to-M1 repolarisation, delivering therapeutic agents (e.g., siRNA or small-molecule inhibitors), and activating immune-related signalling pathways, can reduce immunosuppression and enhance anti-tumour responses [48]. (**B**) T cell-targeting nanoparticles: (**i**) anti-Thy 1.1 or anti-CD45 conjugated liposomes for T cell targeting, and (**ii**) mean fluorescence of pmel-1 Thy1.1+ T cells after co-incubation with targeted liposomes, analysed by flow cytometry ([93], copyright permission by American Chemical Society 2017), (**iii**) anti-CD4 functionalised lipid nanoparticles for siRNA delivery to T cells, and (**iv**) percentage of CD45-silenced CD4+ T cells in blood 5 days after intravenous injection into mice treated with saline (mock), LNPs (siCD45), targeted LNPs (siCD45), isotype LNPs (siCD45), or targeted LNPs with control siRNA; mean ± SD, n = 5, ** *p* <0.005 ([94], copyright permission by American Chemical Society 2015).

Immunomodulatory nanoparticles are also integrated with conventional drug delivery strategies to achieve synergistic therapeutic effects. Co-delivery platforms can combine chemotherapeutic agents with immunomodulators, enabling simultaneous tumour cell killing and immune activation. In addition, stimuli-responsive nanocarriers can release immunotherapeutic payloads in response to tumour-specific cues such as pH, redox conditions, or reactive oxygen species, further improving targeting precision. These combined approaches have been shown to overcome tumour resistance mechanisms, reduce hypoxia, and enhance both innate and adaptive immune responses, thereby improving treatment outcomes [90,91]. Tumour-associated macrophages play a major role in shaping the immunosuppressive tumour microenvironment and represent a key target for nanoparticle-based immunotherapy. Biomaterial-based nanocarriers can selectively interact with these cells and modulate their phenotype, promoting a shift from tumour-supportive to tumour-suppressive phenotypes. Such strategies support both direct therapeutic effects and broader immune activation within the tumour microenvironment. Figure 5A illustrates nanoparticle-mediated regulation of macrophage polarisation and associated signalling pathways to enhance anti-tumour immunity [48]. Figure 5B presents nanoparticles engineered to target T cells, highlighting strategies for delivering immunomodulatory agents such as siRNA or inhibitors to enhance T cell activation. The diagram also depicts T cell infiltration into target tissues and modulation of immune checkpoints, providing a visual summary of T cell-focused nanoparticle immunotherapy mechanisms [92,93,94].

## 5. Precision Nanomedicine

Drawing on advances in targeted delivery and immunomodulation, precision nanomedicine aims to tailor nanoparticle-based therapies to the biological features of individual tumours, moving beyond one-size-fits-all approaches. Tumours differ widely between patients in terms of genetic mutations, microenvironmental conditions, and immune composition, all of which influence drug delivery and therapeutic efficacy. Nanoparticles offer advantages in this context, as their size, surface chemistry, and drug release profiles can be engineered to match specific tumour characteristics. For example, targeted nanoparticles can exploit tumour-specific receptors or microenvironmental cues such as acidity or hypoxia to improve drug accumulation and therapeutic efficacy [95,96].

Another aspect of precision nanomedicine is the integration of diagnostic and therapeutic functions within a single platform. These theranostic systems allow real-time monitoring of nanoparticle distribution and treatment response, supporting more informed clinical decision-making. Advances in biomaterials design have made it possible to incorporate imaging agents alongside therapeutic payloads, supporting patient stratification and treatment optimisation strategies. Such approaches are particularly valuable in cancer, where heterogeneous tumour regions may respond differently to therapy [97,98]. Despite these advances, translation of precision nanomedicine into clinical practice remains challenging. Variability in tumour biology, limited predictive models, and differences between preclinical and human systems can influence therapeutic outcomes. As a result, interest is growing in developing more representative experimental platforms that better capture patient-specific tumour features. These platforms, including organoids, spheroids, and ex vivo systems, are increasingly used to evaluate nanoparticle behaviour and optimise drug delivery strategies in a patient-specific context [99,100].

### 5.1. Patient-Derived Platforms

#### 5.1.1. Organoids

Organoids are three-dimensional cell culture systems derived from patient tissues that can reproduce key structural and functional features of native tumours. Unlike traditional cell lines, organoids preserve aspects of tumour heterogeneity, including genetic mutations and cellular diversity, making them useful for studying nanoparticle delivery and therapeutic response. In cancer research, organoids have been used to evaluate how nanoparticles penetrate tumour tissue, release drugs, and interact with different cell populations under conditions that closely mimic the in vivo environment [101,102]. In the context of drug delivery, organoids provide a platform to assess treatment efficacy and optimise nanoparticle design. For example, studies have shown that nanoparticle penetration and drug release can vary significantly depending on organoid size, density, and extracellular matrix composition. This allows researchers to test different nanoparticle formulations and identify formulations with improved distribution and therapeutic outcomes. Importantly, patient-derived organoids have also been used to predict clinical responses to therapy, supporting their potential role in guiding personalised treatment strategies [103,104].

#### 5.1.2. Spheroids

Tumour spheroids are simpler three-dimensional models formed from cancer cell lines or primary cells, and they are widely used for studying nanoparticle transport and drug penetration. Although less complex than organoids, spheroids can reproduce key physical features of solid tumours, such as diffusion gradients, hypoxia, and cell–cell interactions. These characteristics make them useful for evaluating how nanoparticles move through dense tumour tissue and how effectively drugs are released within different tumour regions [105,106].

Spheroids are useful for investigating barriers to drug delivery, such as limited penetration into the tumour core. Nanoparticles often accumulate at the outer layers of spheroids, with reduced diffusion into inner regions mirroring challenges observed in solid tumours. By using spheroid models, researchers can systematically test strategies to improve penetration, such as modifying nanoparticle size, surface charge, or responsiveness to tumour-specific stimuli. Spheroids, therefore, remain an important and accessible tool for optimising nanoparticle-based drug delivery systems [107,108].

#### 5.1.3. Ex Vivo Systems

Ex vivo tumour models involve the use of freshly resected patient tissues that are maintained outside the body while preserving their native architecture and cellular composition. These systems retain complex interactions between tumour cells, stromal components, and immune cells, providing a physiologically relevant environment for studying nanoparticle behaviour. Compared to in vitro models, ex vivo platforms more closely approximate clinical conditions, making them particularly valuable for evaluating drug delivery and therapeutic response [109,110].

In nanoparticle research, ex vivo systems have been used to assess tissue penetration, cellular uptake, and immune interactions in a patient-specific context. For example, these models can reveal how nanoparticles distribute within heterogeneous tumour regions or how immune cells respond to nanoparticle-mediated therapies. Such detail is difficult to achieve with conventional models and can provide insights for optimising treatment strategies. As precision nanomedicine continues to evolve, ex vivo platforms are likely to play an increasingly important role in bridging laboratory studies and clinical application [104,111]. Such patient-derived platforms provide complementary advantages for evaluating nanoparticle-based drug delivery in cancer. Each model captures different aspects of tumour biology, from structural complexity to microenvironmental interactions, influencing nanoparticle transport and therapeutic response. Table 6 provides a comparative overview of these systems, summarising their key features, applications, and limitations in nanomedicine research.

**Figure 6:**
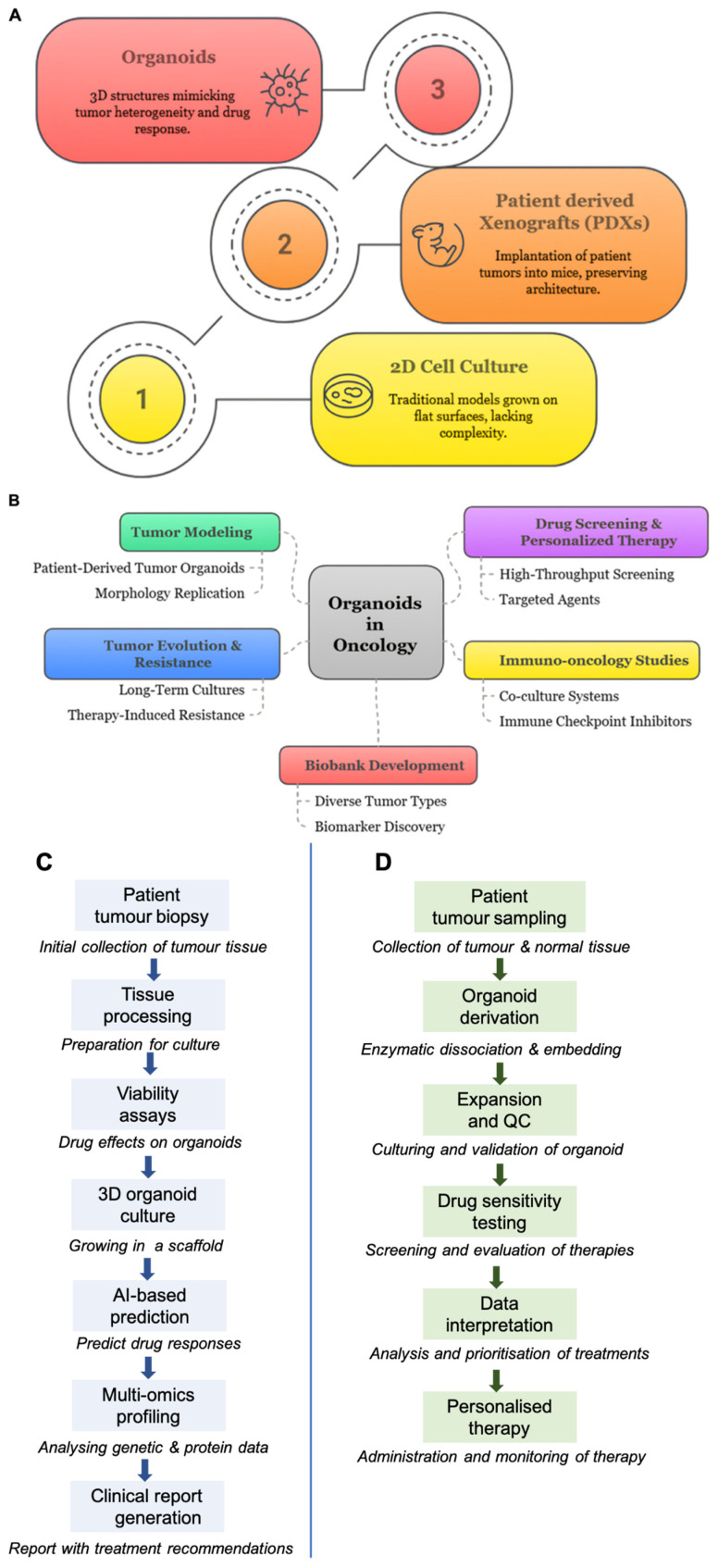
Patient-derived organoids as advanced platforms for cancer modelling and precision therapy. (**A**) Evolution of cancer models from two-dimensional (2D) cell cultures to in vivo systems and three-dimensional organoids, highlighting increasing physiological relevance and tumour complexity. (**B**) Multifunctional applications of tumour organoids in oncology, including tumour modelling, drug screening, immune oncology, resistance studies, and biobanking. (**C**) Workflow of patient-derived organoid-based drug testing, from tumour tissue processing to high-throughput screening and therapeutic response profiling. (**D**) Clinical integration of organoid platforms, illustrating the use of patient-derived tumour organoids for personalised therapy selection and treatment optimisation. Adapted from [112].

Cancer models have evolved in response to the need for more physiologically relevant systems. Early approaches, such as two-dimensional (2D) cell cultures, provided simple and controllable platforms for studying cancer biology but failed to capture the structural complexity, cellular diversity, and microenvironmental interactions of tumours in vivo. More advanced models, including patient-derived xenografts (PDXs) and genetically engineered mouse models (GEMMs), improved biological relevance but remain limited by high cost, long development times, and restricted scalability. This has led to the emergence of three-dimensional organoid models, which better recapitulate tumour architecture and heterogeneity while offering greater potential for patient-specific applications [112,113,114] (Figure 6A).

Patient-derived organoids (PDOs), generated from tumour biopsies or surgical specimens, retain key characteristics of the original tumour, including genetic alterations, phenotypic diversity, and spatial organisation. This makes them valuable for studying tumour behaviour and therapeutic response in a more clinically relevant context. As shown in Figure 6B, organoids support a wide range of applications in oncology, including tumour modelling, drug screening, investigation of resistance mechanisms, and immune-oncology studies. They also bridge the gap between conventional in vitro and in vivo models, supporting more accurate and reproducible evaluation of treatment strategies [112,115,116,117,118,119].

The integration of PDOs into drug development and clinical workflows represents an important step toward functional precision medicine. Organoid-based platforms enable rapid and scalable testing of therapeutic agents, supporting the identification of patient-specific treatment responses. Figure 6C illustrates a typical workflow, from tumour dissociation to high-throughput drug screening and response profiling. These approaches are increasingly being translated into clinical settings, where organoid testing may inform treatment decisions in near real time. This includes the generation of organoids from patient samples, followed by drug testing and personalised therapy selection, demonstrating the growing role of PDOs in bridging experimental research and clinical oncology [112,120,121] (Figure 6D).

### 5.2. Data-Driven and AI-Enabled Precision Nanomedicine

The integration of artificial intelligence (AI) and data-driven approaches is reshaping the design and optimisation of nanoparticle-based therapies. Traditional nanomedicine development has relied largely on trial-and-error experimentation, which can be time-consuming and inefficient given the complexity of tumour biology and nanoparticle interactions. AI offers an alternative approach by analysing large datasets and identifying patterns that are not easily captured through conventional methods. Machine learning algorithms can process information on nanoparticle size, surface chemistry, and biological interactions to guide the rational design of more effective drug delivery systems. This shift toward data-driven design is accelerating the development of nanomedicines while improving their precision and reproducibility [122,123].

A major advantage of AI in nanomedicine is its ability to model complex nanoparticle–tumour interactions across multiple biological scales. The behaviour of nanoparticles in the body depends on both physicochemical properties and biological factors, including protein corona formation, cellular uptake, and tissue distribution. AI-driven models can integrate these variables to predict nanoparticle behaviour in vivo, including their bio-distribution, circulation time, and therapeutic efficacy. For example, computational models trained on large datasets have been shown to predict nanoparticle delivery efficiency to tumours with high accuracy (*R*^2^ values up to ~0.83), where *R*^2^ represents the proportion of variance in the data explained by the model, with values closer to 1 indicating better predictive performance, demonstrating their potential to guide formulation design before experimental testing [124]. More recent studies also report predictive accuracies ranging from 90 to 99% in analysing nanoparticle–cell interactions, supporting the growing reliability of these approaches [125].

Beyond prediction, AI is increasingly used to design and optimise nanoparticle systems. Advanced techniques such as deep learning and generative modelling allow researchers to screen thousands of nanoparticle formulations in silico and identify the most promising candidates. These approaches can optimise key parameters such as drug loading, release kinetics, and targeting efficiency, significantly reducing the need for extensive experimental screening. In addition, AI can analyse imaging and high-throughput screening data to uncover relationships between nanoparticle structure and biological response, enabling more informed decision-making during development. This is particularly valuable in cancer research, where small changes in the tumour microenvironment can significantly influence treatment outcomes [126,127].

AI-driven nanomedicine is also moving toward personalised optimisation by incorporating patient-specific data into predictive models. By analysing genomic, proteomic, and tumour microenvironment data, AI systems can predict nanoparticle designs tailored to individual patients, improving therapeutic efficacy while reducing off-target effects. For example, machine learning models can predict how specific tumour characteristics will influence nanoparticle uptake and drug response, supporting more precise treatment selection. These approaches are particularly useful when combined with patient-derived platforms, such as organoids, which generate high-quality experimental data for model training and validation. As these technologies continue to evolve, AI-enabled nanomedicine is expected to play an important role in advancing truly personalised cancer therapy [125,128].

#### 5.2.1. Predictive Modelling of Nanoparticle–Tumour Interactions

Understanding how nanoparticles behave within tumours remains a central challenge in cancer drug delivery. Their distribution is influenced not only by particle size and surface chemistry but also by tumour-specific factors such as vascular permeability, extracellular matrix density, and immune cell composition. Predictive modelling provides a way to integrate these variables and anticipate how nanoparticles will perform before extensive experimental testing. Early work demonstrated that only a small fraction of administered nanoparticles, often less than 1%, reaches tumour tissue, highlighting the need for more efficient design strategies [129].

Recent advances in machine learning have improved the ability to predict nanoparticle delivery and therapeutic outcomes. By training algorithms on large experimental datasets, researchers can identify patterns linking nanoparticle properties to biological responses such as tumour accumulation, cellular uptake, and drug release. For example, models developed using physicochemical descriptors and biological parameters have achieved strong predictive performance for tumour delivery efficiency (*R*^2^ values approaching ~0.8), demonstrating that computational tools can help guide nanoparticle design. These approaches reduce reliance on trial-and-error experimentation and allow prioritisation of promising formulations for further validation [130]. Emerging computational frameworks also aim to capture the dynamic interactions between nanoparticles and the tumour microenvironment. These include modelling protein corona formation, immune recognition, and transport across biological barriers, all of which influence therapeutic efficacy. Integration of such multiscale data remains challenging, but advances in data availability and computational power are enabling more realistic simulations. As these models continue to evolve, they are expected to help bridge the gap between preclinical studies and clinical translation of nanomedicine [131].

#### 5.2.2. Personalised Optimisation Using Patient-Derived Data

While predictive modelling provides broader design principles, incorporating patient-specific data is essential for achieving true precision nanomedicine. Tumours vary widely between individuals in terms of genetic mutations, microenvironmental features, and immune composition, all of which influence nanoparticle delivery and treatment response. By integrating genomic, proteomic, and imaging data, computational models can be adapted to predict how an individual patient’s tumour will respond to a given nanoparticle formulation. This approach supports more informed therapy selection and may reduce ineffective treatment [132].

**Figure 7:**
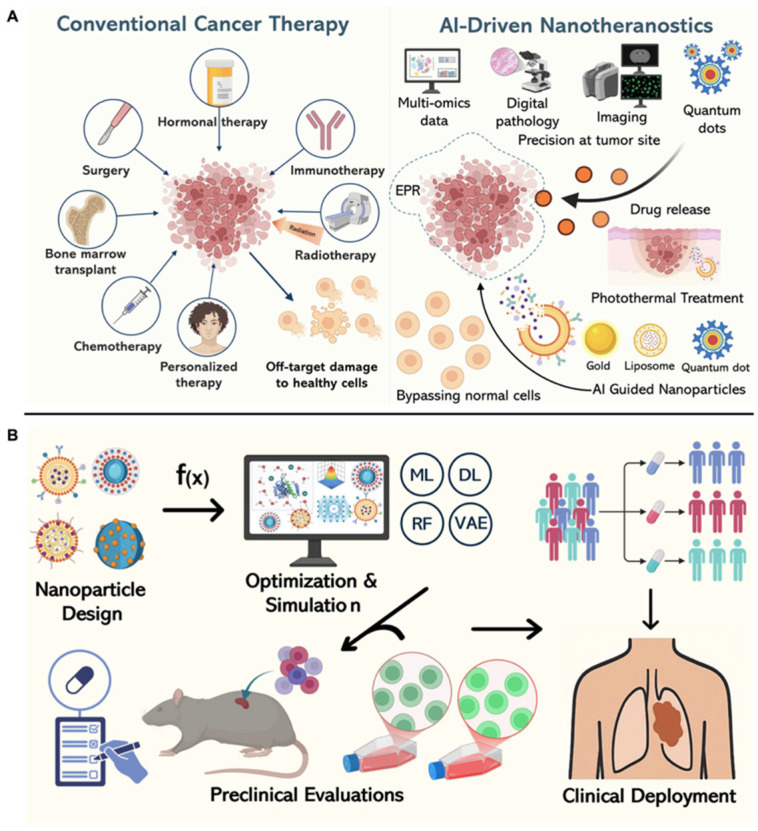
AI-driven nanomedicine for precision cancer therapy. (**A**) Comparison between conventional cancer therapies and AI-guided nano-theranostics, highlighting improved targeting specificity, reduced off-target toxicity, and integration of multi-omics and imaging data. (**B**) Workflow of AI-driven nanoparticle design, illustrating the use of machine learning and deep learning approaches for nanoparticle optimisation, preclinical validation, and clinical translation toward personalised cancer treatment. Adapted from [126]. ML, machine learning; DL, deep learning; RF, Random Forest (a machine learning algorithm); VAE, variational autoencoder.

The combination of AI-driven modelling with patient-derived experimental platforms further strengthens personalised approaches. Data generated from organoids, spheroids, or ex vivo tumour samples can be used to train and validate predictive models, creating an iterative feedback loop between computation and experimentation. For instance, drug response data from patient-derived organoids have been shown to correlate with clinical outcomes, supporting their value in guiding treatment decisions. When integrated with AI tools, these platforms enable rapid optimisation of nanoparticle formulations for individual patients, bringing cancer therapy closer to real-time, data-driven optimisation [133]. Artificial intelligence is increasingly integrated throughout the nanomedicine pipeline, from initial nanoparticle design to clinical implementation. AI-driven approaches support improved targeting precision and data-guided optimisation of therapeutic strategies. Figure 7 summarises how these technologies are reshaping cancer treatment, from nanoparticle design to translational workflows. While these advances highlight the potential of precision nanomedicine, their successful clinical implementation depends on overcoming a range of translational, manufacturing, regulatory, and safety challenges. These considerations are discussed in the following section.

## 6. Translation and Clinical Outlook

Despite substantial advances in nanoparticle engineering and precision nanomedicine, the clinical translation of cancer nanomedicine has progressed more slowly than initially anticipated. Many nanoparticle systems that show promising results in preclinical studies fail to achieve similar outcomes in patients, largely because tumour biology in humans is considerably more complex and heterogeneous than in experimental models. Factors such as variable vascular permeability, immune clearance, protein corona formation, and differences in the tumour microenvironment can significantly alter nanoparticle distribution and therapeutic response. These limitations highlight the need for more predictive experimental systems and better patient stratification strategies during early-stage development [134,135].

At the same time, several nanoparticle-based therapies have successfully entered clinical practice, demonstrating the translational potential of nanomedicine platforms when formulation design and clinical need are well aligned. Liposomal formulations such as Doxil and albumin-bound nanoparticles such as Abraxane remain among the best-known examples, improving pharmacokinetics and reducing systemic toxicity compared with conventional chemotherapy. More recently, advances in biomaterials design, targeting ligands, and stimuli-responsive systems have expanded the therapeutic scope of nanoparticle platforms beyond passive drug delivery. Current clinical trials increasingly explore multifunctional nanoparticles for combination therapies, immunomodulation, and image-guided treatment approaches [136,137].

The future clinical impact of nanomedicine will likely depend on stronger integration between materials science, computational modelling, and patient-specific biology. Researchers are increasingly combining nanoparticle engineering with artificial intelligence, organoid platforms, and molecular profiling to identify formulations that are more likely to be effective in individual patients. This reflects a broader shift toward precision oncology, where therapeutic design is informed not only by tumour type but also by dynamic biological and microenvironmental features. As these approaches mature, the field is gradually moving from proof-of-concept nanocarriers toward clinically adaptable and reproducible therapeutic systems [138].

### 6.1. Bench to Bedside

A major barrier to clinical translation is the discrepancy between preclinical nanoparticle performance and therapeutic outcomes in humans. Many studies rely on simplified tumour models that do not fully reproduce the complexity of human cancers, including stromal interactions, immune regulation, and interpatient heterogeneity. As a result, nanoparticles that demonstrate efficient tumour accumulation in animal models often show limited delivery efficiency in clinical settings. A widely cited analysis reported that less than 1% of injected nanoparticles reach solid tumours, highlighting the need for improved delivery strategies and more predictive evaluation platforms [134].

Manufacturing and scalability remain major challenges during translation from laboratory research to clinical application. Nanoparticle formulations must maintain consistent physicochemical properties, drug loading efficiency, and stability across large-scale production batches. Small variations in synthesis methods can alter bio-distribution, toxicity, or therapeutic performance. Regulatory agencies, therefore, require rigorous characterisation and reproducibility before clinical approval. These challenges are amplified for multifunctional or stimuli-responsive nanoparticles, where structural complexity can complicate quality control and regulatory assessment [136,139].

Despite these challenges, the number of nanomedicine-related clinical trials is increasing. Current trials are evaluating targeted nanoparticles for chemotherapy delivery, RNA therapeutics, cancer immunotherapy, and image-guided treatment. Several lipid nanoparticle platforms developed for nucleic acid delivery have also increased interest in clinically translatable nanocarriers, particularly following the success of mRNA-based technologies [55]. Importantly, recent clinical strategies increasingly incorporate biomarker profiling and patient stratification, reflecting growing recognition that nanoparticle efficacy is strongly influenced by tumour-specific biological factors [140,141].

In addition to lipid nanoparticles, several next-generation gene delivery platforms are increasingly relevant to cancer nanomedicine, including EVs and viral vectors such as AAVs. EVs, including exosomes, are naturally derived vesicles with intrinsic biocompatibility and cell-targeting capabilities, making them attractive for RNA and protein delivery. However, challenges remain in scalable production, cargo loading efficiency, and purification standardisation. AAV vectors remain among the most efficient gene delivery systems and have demonstrated durable transgene expression in clinical settings, but their application is limited by pre-existing immunity, restricted payload capacity, and high manufacturing complexity. Overall, these platforms highlight that gene delivery is no longer dominated by a single modality but instead represents a spectrum of synthetic, biological, and viral systems, each with distinct translational advantages and limitations [142].

### 6.2. Regulatory and Ethical Issues

As nanomedicine platforms become increasingly sophisticated, regulatory evaluation has become increasingly complex. Unlike conventional small-molecule drugs, nanoparticle systems often combine multiple functional components, including targeting ligands, imaging agents, and stimuli-responsive materials. This complexity can make it difficult to establish standardised characterisation methods or directly compare results across studies. Parameters such as particle size distribution, surface charge, stability, and protein corona formation can all influence biological behaviour, yet these features are not always measured consistently between laboratories. Improving standardisation and reproducibility is essential for ensuring reliable safety assessment and clinical translation [143,144]. From a regulatory perspective, emerging gene delivery platforms such as ionisable LNPs, EVs, and AAV vectors introduce additional layers of complexity. LNPs require careful control of composition-dependent variability, biodistribution, and repeat-dose immunogenicity. EV-based therapeutics face regulatory challenges related to product heterogeneity, donor variability, and lack of standardised manufacturing and characterisation pipelines. Viral vectors such as AAVs are subject to stringent safety evaluation due to concerns regarding immunogenicity, insertional risk, and long-term persistence. These challenges underscore the need for harmonised regulatory frameworks capable of accommodating both synthetic and biologically derived delivery systems in clinical translation [55,142].

Reproducibility has also become a broader concern in preclinical nanomedicine research. Variations in experimental design, cell models, animal models, and reporting practices can affect therapeutic outcomes and complicate comparisons between studies. Several groups have emphasised the need for harmonised protocols, transparent reporting standards, and better integration of clinically relevant models during nanoparticle evaluation. Efforts to establish standardised testing frameworks are increasingly considered necessary not only for regulatory approval but also for improving confidence in nanomedicine research across academia and industry [145,146].

The rise in personalised nanomedicine also raises important ethical considerations. AI-guided therapeutic design, molecular profiling, and patient-derived platforms rely heavily on sensitive biological and clinical data, raising concerns about privacy, data ownership, and equitable access to advanced therapies. Highly individualised nanomedicine strategies may increase treatment costs and widen disparities between healthcare systems with differing technological resources. Ethical implementation will require careful balancing of innovation, patient benefit, transparency, and accessibility. As precision nanomedicine continues to evolve, ethical and regulatory frameworks will need to evolve alongside scientific advances to support safe and equitable clinical translation [147,148]. Despite major advances in nanoparticle engineering, scientific, clinical, and regulatory challenges continue to limit the widespread clinical translation of cancer nanomedicine. These barriers include issues related to biological variability, manufacturing reproducibility, long-term safety, and patient-specific treatment responses. Table 7 summarises the major translational and regulatory considerations shaping clinical development of nanoparticle-based cancer therapies.

From a platform-specific perspective, the translational challenges of gene delivery systems vary significantly across different technologies discussed in this review. For ionisable LNPs, key limitations include off-target biodistribution (particularly hepatic accumulation), dose-dependent innate immune activation, and challenges associated with repeat administration due to anti-PEG or lipid-associated immune responses, despite their strong scalability and clinical validation. EVs face distinct barriers related to scalable and reproducible manufacturing, heterogeneity in vesicle populations, limited cargo loading efficiency, and the absence of universally standardised purification and characterisation methods, which together hinder clinical translation. AAV vectors offer highly efficient and durable gene transfer but are constrained by pre-existing neutralising immunity, restricted packaging capacity, potential hepatotoxicity at high doses, and high manufacturing costs [55,142]. Collectively, these platform-specific considerations highlight that the clinical success of gene delivery in cancer therapy depends not only on payload design but also on overcoming distinct engineering, immunological, and manufacturing barriers inherent to each delivery modality.

## 7. Conclusions and Future Perspectives

Nanoparticle-based biomaterials have substantially expanded cancer research and therapy, moving beyond conventional drug carriers toward multifunctional platforms that can probe tumour biology, overcome therapeutic resistance, and enable precision treatment strategies. Throughout this review, nanoparticles were discussed not only as delivery systems but also as mechanistic tools that provide insight into tumour heterogeneity, immune regulation, and tumour microenvironmental dynamics. Their ability to respond to biochemical and biophysical cues, selectively accumulate within diseased tissues, and integrate therapeutic and diagnostic functions has positioned nanomedicine at the intersection of biomaterials science, oncology, and advanced drug delivery. The field is also undergoing a broader conceptual shift. Earlier nanomedicine strategies focused primarily on improving pharmacokinetics or reducing systemic toxicity; current approaches increasingly focus on adaptive and biologically informed systems capable of interacting with complex tumour ecosystems. Advances in stimuli-responsive nanoparticles, immunomodulatory biomaterials, and multifunctional theranostic platforms illustrate the shift toward more sophisticated and clinically relevant applications. In parallel, patient-derived models such as organoids, spheroids, and ex vivo systems are improving the evaluation of nanoparticle performance under conditions that more closely resemble human disease. These platforms provide opportunities to investigate treatment response, tumour evolution, and resistance mechanisms in a patient-specific context.

The integration of artificial intelligence and computational modelling further supports this transition toward precision nanomedicine. Data-driven approaches are increasingly being used to predict nanoparticle–tumour interactions, optimise formulation design, and support personalised therapeutic selection using patient-derived biological data. Although these technologies remain at relatively early stages of implementation, they offer potential solutions to longstanding challenges related to variability in nanoparticle delivery and treatment efficacy. Combining computational tools with clinically relevant experimental models may help reduce the translational gap that has historically limited the success of cancer nanomedicine. Despite considerable progress, several barriers continue to limit widespread clinical adoption. Reproducibility, scalable manufacturing, regulatory standardisation, and long-term safety assessment remain significant challenges, particularly for complex multifunctional nanoplatforms. Biological variability between patients and tumour subtypes complicates the identification of universally effective nanoparticle formulations. Addressing these limitations will require closer collaboration between biomaterials science, cancer biology, computational modelling, regulatory science, and clinical oncology. Future work should prioritise robust validation in clinically relevant models, transparent reporting standards, and the development of adaptable nanomedicine platforms that can accommodate patient-specific disease characteristics.

Overall, the future of cancer nanomedicine may depend less on increasingly complex nanoparticles alone and more on the intelligence of biomaterials science with precision biology, advanced modelling, and translational medicine. As experimental, computational, and clinical approaches become more interconnected, nanoparticle-based systems are expected to play an increasingly important role in developing safer, more targeted, and more personalised cancer therapies.

## Figures and Tables

**Table 1:** Nanoparticles as mechanistic probes of cancer cell-ECM interactions and uptake.

Nanoparticle Type	Key Tunable Properties	Biological Process Probed	Mechanistic Insight Identified	Ref.
Polymeric nanoparticles (e.g., PLGA, PEG-based)	Size, surface charge, ligand density	ECM adhesion and integrin engagement	Demonstrated nanoscale ligand control of adhesion and migration	[16,24]
Liposomes	Lipid composition, surface functionalisation	Cellular uptake and endocytosis	Revealed size and charge-dependent uptake pathways and intracellular trafficking	[17]
Gold nanoparticles (AuNPs)	Size, shape, surface chemistry	Mechanotransduction and receptor clustering	Enabled probing of force-sensitive signalling and membrane receptor organisation	[25]
Mesoporous silica nanoparticles	Pore size, surface modification	ECM diffusion and penetration	Quantified how ECM density and composition limit nanoparticle transport	[26]
Iron oxide nanoparticles (SPIONs)	Core size, magnetic properties	Uptake dynamics and intracellular fate	Provided insight into endocytic routes and spatial heterogeneity in uptake	[22,23]
Quantum dots	Size, surface coating, fluorescence	Tracking uptake and intracellular localisation	Enabled real-time visualisation of nanoparticle trafficking and cell-to-cell variability	[17,23]

**Table 2:** Summary of nanoparticle-based approaches used to investigate and address tumour heterogeneity and therapy resistance.

Barrier/Feature	NP Strategy	Mechanistic Insight or Therapeutic Role	Representative NP Type	Ref.
Hypoxia gradients	Hypoxia-responsive NPs	Selective activation in hypoxic tumour regions	Bioreductive polymeric NPs	[45]
Dense ECM	ECM-penetrating NPs	Enhanced tumour penetration and ECM navigation	Collagenase-functionalised NPs	[40]
Cellular heterogeneity	Targeted NPs	Target resistant tumour subpopulations	Antibody/aptamer-functionalised NPs	[46]
Drug efflux/MDR	Co-delivery NPs	Circumvention multidrug resistance mechanisms	Chemosensitiser-loaded NPs	[47]
Immunosuppressive niches	Immunomodulatory NPs	Modulate immunosuppressive tumour niches	Macrophage-targeting NPs	[48]
Therapy-induced resistance	Multi-responsive NPs	Adaptive response to dynamic tumour conditions	Smart responsive NPs	[49]

CSC, cancer stem cells; TAM, tumour-associated macrophages; MDR, multidrug resistance; CSC-like cells, tumour cells exhibiting stem cell-like properties (e.g., self-renewal, therapy resistance).

**Table 3:** Major nanoparticle platforms used for cancer drug delivery and targeting.

Nanoparticle Platform	Structural Characteristics	Key Advantages for Cancer Therapy	Representative Applications	Ref.
Liposomes	Phospholipid bilayer vesicles capable of encapsulating hydrophilic and hydrophobic drugs	Excellent biocompatibility, clinically validated systems, improved pharmacokinetics and reduced systemic toxicity	Delivery of chemotherapeutics (e.g., doxorubicin formulations), targeted liposomal therapies (NOT including nucleic acid delivery systems)	[59]
Ionisable Lipid Nanoparticles (LNPs)	Ionisable lipid, helper phospholipid, cholesterol, PEG-lipid	pH-responsive ionisation enabling efficient RNA encapsulation and endosomal escape, clinically validated for nucleic acid delivery	siRNA/mRNA delivery systems for cancer immunotherapy and gene-editing therapeutics	
Polymeric Nanoparticles	Biodegradable polymers such as PLGA, PEG, or PLA forming nano-spheres or nano-capsules	Controlled drug release, tunable degradation, versatile surface modification for targeting	Delivery of chemotherapy drugs, siRNA, or combination therapies	[60]
Dendrimers	Highly branched, tree-like macromolecules with multiple terminal functional groups	Precise molecular architecture, high drug loading, multiple surface modification sites	Targeted delivery of anticancer drugs and nucleic acids	[61]
Gold Nanoparticles	Metallic nanoparticles with tunable size and optical properties	Easy surface functionalisation, imaging capability, photothermal therapy potential	Drug delivery combined with photothermal or imaging applications	[62]
Mesoporous Silica Nanoparticles	Porous inorganic nanoparticles with large internal surface area	High drug loading capacity, controlled release, stable structure	Delivery of chemotherapeutics and combination therapies	[63]
Magnetic (Iron Oxide) Nanoparticles	Superparamagnetic nanoparticles responsive to external magnetic fields	Magnetic targeting, imaging capability (MRI), potential for hyperthermia therapy	Targeted drug delivery and magnetic-guided tumour therapy	[64]

**Table 4:** Common targeting ligands used for nanoparticle-based drug delivery in cancer.

Targeting Ligand	Target Receptor/Biomarker	Nanoparticle Type	Therapeutic Purpose	Ref.
Folic acid	Folate receptor (overexpressed in ovarian, breast, lung cancers)	Polymeric NPs, liposomes	Enhances tumour-specific uptake through receptor-mediated endocytosis	[65,66]
Monoclonal antibodies (e.g., anti-HER2)	HER2 receptor	Liposomes, polymeric NPs	Targeted delivery to HER2-positive tumours	[60]
RGD peptides	Integrin α_v_β_3_ (angiogenic tumour vasculature)	Polymeric and inorganic NPs	Targeting tumour angiogenesis and improving nanoparticle penetration	[67]
Aptamers (e.g., AS1411)	Nucleolin and other tumour-associated proteins	Gold NPs, polymeric NPs	Highly specific binding and targeted drug delivery	[68]
Transferrin	Transferrin receptor (highly expressed in rapidly dividing cancer cells)	Liposomes, polymeric NPs	Improved uptake in tumour cells with high iron demand	[69,70]
Hyaluronic acid	CD44 receptor (tumour cells and cancer stem cells)	Polymeric and lipid nanoparticles	Targeting tumour cells and tumour microenvironment	[71,72,73]

**Table 5:** Smart and multifunctional nanoparticle strategies in cancer therapy.

Strategy	Trigger/Mechanism	Example System	Key Outcome	Ref.
pH-responsive NP	Acidic tumour microenvironment	DOX-loaded nanogels	Enhanced tumour-specific drug release and cytotoxicity	[76]
Redox-responsive NP	High glutathione (GSH)	Disulfide-linked nanoparticles	Triggered intracellular drug release	[77]
Dual pH/redox NP	Combined stimuli	Polymeric nanocarriers	Improved uptake and therapeutic efficacy	[79]
Enzyme/multi-responsive NP	Enzymes + pH/redox	Hybrid nanocarriers	Enhanced intracellular delivery	[78]
ROS-responsive NP	Oxidative stress	ROS-sensitive nanoplatforms	Selective tumour activation	[80]
Theranostic NP	Imaging + therapy	Redox-responsive nanoplatforms	Simultaneous diagnosis and treatment	[81]
MDR-targeting NP	Efflux bypass/redox modulation	Redox-manipulating nanocarriers	Increased drug sensitivity	[87]

**Table 6:** Patient-derived platforms for nanoparticle evaluation in cancer.

Model	Key Features	Application in Nanomedicine	Advantages	Limitations	Ref.
Organoids	3D, patient-derived, heterogeneous	Drug screening, NP penetration	High clinical relevance	Complex, costly	[101,103]
Spheroids	3D, simpler structure	NP transport, diffusion studies	Reproducible, scalable	Less heterogeneity	[105,107]
Ex vivo tissues	Native tumour architecture	NP distribution, immune response	Highly realistic	Limited lifespan	[109,110]

**Table 7:** Key translational, regulatory, and clinical challenges in cancer nanomedicine.

Challenge	Impact on Clinical Translation	Current Strategies	Representative Examples/Focus	Ref.
Limited tumour accumulation	Reduced therapeutic efficacy	Active targeting, microenvironment-responsive systems	Ligand-functionalised NPs (e.g., antibody/peptide-modified systems), LNP-based delivery	[134]
Manufacturing variability	Poor batch reproducibility	Standardised synthesis and GMP-compatible production	LNPs, polymeric NPs, extracellular vesicle (EV) manufacturing pipelines	[139]
Biological heterogeneity	Variable patient response	Patient-derived models, biomarker stratification, AI-guided selection	Organoids, EV-based biomarkers, personalised nanomedicine platforms	[138]
Regulatory complexity	Delayed approval pathways	Harmonised characterisation protocols, platform-specific regulatory frameworks	FDA/EMA nanomedicine guidelines, gene therapy (AAV), LNP-based therapeutics	[143]
Long-term safety concerns	Uncertain toxicity and immunotoxicity studies	Longitudinal biodistribution and immunotoxicity studies	Inorganic NPs, ionised LNPs, AAV vectors (dose-dependent toxicity considerations)	[146]
Ethical considerations	Inequitable access and data governance concerns	Ethical AI governance, transparency, equitable clinical implementation	Personalised nanomedicine, AI-guided therapy selection, gene therapy platforms	[147]

NPs, nanoparticles; GMP, good manufacturing practice; FDA, Food and Drug Administration; EMA, European Medicines Agency.

## Data Availability

No new data were created or analysed in this study. Data sharing is not applicable to this article.

## Abbreviations

AI	Artificial intelligence
AKT	Protein kinase B
AuNPs	Gold nanoparticles
CSC	Cancer stem cells
CT	Computed tomography
CRISPR	Clustered regularly interspaced short palindromic repeats
CD44	Cluster of differentiation 44 (cell-surface glycoprotein receptor)
DL	Deep learning
ECM	Extracellular matrix
ERK	Extracellular signal-regulated kinase
EPR	Enhanced permeability and retention
EMA	European Medicines Agency
FDA	Food and Drug Administration
FAK	Focal adhesion kinase
GEMMs	Genetically engineered mouse models
GMP	Good manufacturing practice
GSH	High glutathione
HIFs	Hypoxia-inducible factors
IBR	Ibrutinib (Bruton’s tyrosine kinase inhibitor)
IFN-γ	Interferon gamma (pro-inflammatory cytokine involved in immune activation)
IL-12	Interleukin-12 (cytokine that promotes T cell and NK cell activation)
LOX	Lysyl oxidase
MDSCs	Myeloid-derived suppressor cells
MDR	Multidrug resistance
MRI	Magnetic resonance imaging
ML	Machine learning
MMPs	Matrix metalloproteinases
mRNA	Messenger RNA (messenger ribonucleic acid)
NPs	Nanoparticles
PDOs	Patient-derived organoids
PDGFR	Platelet-derived growth factor receptor
PDXs	Patient-derived xenografts
PEG	Polyethene glycol
PLGA	Poly (lactic-co-glycolic acid)
PLA	Poly (lactic acid)
Regorafenib	Multi-kinase inhibitor targeting angiogenic and oncogenic pathways
RF	Random forest (a machine learning algorithm)
RhoA	Ras homolog family member A
ROCK	Rho-associated kinase
ROS	Reactive oxygen species
SPIONs	Superparamagnetic iron oxide nanoparticles
SRC	SRC proto-oncogene tyrosine kinase
siRNA	Small-interfering RNA
TAM	Tumour-associated macrophage
TAZ	Transcriptional co-activator with PDZ-binding motif
TME	Tumour microenvironment
TGF-β	Transforming growth factor beta
Tregs	Regulatory T cells
VAE	Variational autoencoder
VEGF	Vascular endothelial growth factor
YAP	Yes-associated protein
